# Cluster randomized trial in smoking cessation with intensive advice in diabetic patients in primary care. ITADI Study

**DOI:** 10.1186/1471-2458-10-58

**Published:** 2010-02-04

**Authors:** Lydia Roig, Santiago Perez, Gemma Prieto, Carlos Martin, Mamta Advani, Angelina Armengol, Pilar Roura, Josep Maria Manresa, Elena Briones, ITADI Study Group

**Affiliations:** 1 Basic Area of Health La Garriga, Catalan Institute of Health, (Passeig de l'Ametlla,17),La Garriga, (08480), Spain; 2 Basic Area of Health La Llagosta, Catalan Institute of Health, (Carrer Vic, s/n), La Llagosta, (08120), Spain; 3 Gerencia de Atención Primaria de Ávila, Sanidad de Castilla y Léon, (Avenida Portugal 47), Àvila, (05001), Spain; 4 Basic Area of Health Passeig Sant Joan, Catalan Institute of Health, (Passeig Sant Joan 29), Barcelona, (08010), Spain; 5 Scientific Area, IDIAP Jordi Gol, (Gran Via de les Corts Catalanes 587) Barcelona, (08007), Spain; 6 ABS Terrassa Nord, Catalan Institute of Health, (Carrer Mercè Rodoreda 24) Terrassa, (08207), Spain; 7 ABS Badia, Catalan Institute of Health (Carrer Bètica) , Badia del Vallés, (08214), Spain; 8 USR Metropolitana Nord, IDIAP Jordi Gol, Sabadell, (08203), Spain; 9 USR Barcelona, IDIAP Jordi Gol, (St Elies 42), Barcelona, (08006), Spain

## Abstract

**Background:**

It is a priority to achieve smoking cessation in diabetic smokers, given that this is a group of patients with elevated cardiovascular risk. Furthermore, tobacco has a multiplying effect on micro and macro vascular complications. Smoking abstinence rates increase as the intensity of the intervention, length of the intervention and number and diversity of contacts with the healthcare professional during the intervention increases. However, there are few published studies about smoking cessation in diabetics in primary care, a level of healthcare that plays an essential role in these patients. Therefore, the aim of the present study is to evaluate the effectiveness of an intensive smoking cessation intervention in diabetic patients in primary care.

**Methods/Design:**

Cluster randomized trial, controlled and multicentric. Randomization unit: Primary Care Team. Study population: 546 diabetic smokers older than 14 years of age whose disease is controlled by one of the primary care teams in the study. Outcome Measures: Continuous tobacco abstinence (a person who has not smoked for at least six months and with a CO level of less than 6 ppm measured by a cooximeter) , evolution in the Prochaska and DiClemente's Transtheoretical Model of Change, number of cigarettes/day, length of the visit. Point of assessment: one- year post- inclusion in the study. Intervention: Brief motivational interview for diabetic smokers at the pre-contemplation and contemplation stage, intensive motivational interview with pharmacotherapy for diabetic smokers in the preparation-action stage and reinforcing intevention in the maintenance stage. Statistical Analysis: A descriptive analysis of all variables will be done, as well as a multilevel logistic regression and a Poisson regression. All analyses will be done with an intention to treatment basis and will be fitted for potential confounding factors and variables of clinical importance. Statistical packages: SPSS15, STATA10 y HLM6.

**Discussion:**

The present study will try to describe the profile of a diabetic smoker who receives the most benefit from an intensive intervention in primary care. The results will be useful for primary care professionals in their usual clinical practice.

**Trial Registration:**

Clinical Trials.gov Identifier: NCT00954967

## Background

It is known that tobacco is the principal isolated cause of avoidable morbimortality in developed countries. Half of these deaths are premature, provoking a loss of 5.5 million potential years of life annually, 3.3 million in men and 2.2 million in women [1]. Smoking related diseases are the cause of death in 50% of smokers [2]. The most recent mortality data indicate that tobacco causes 54,233 deaths annually in Spain [3]. In a study about mortality attributable to the active consumption of tobacco in Spain from 1978 to 1992, it was observed that 1 out of every 3 deaths was in a person younger than 65 years of age who had a high life expectancy [3].

The morbimortality observed in diabetics is related to macro and microvascular complications, and tobacco has a multiplying effect on these vascular complications. Furthermore, it has been demonstrated that tobacco consumption is associated with mortality due to isquemic heart disease in diabetic patients [4]. A number of studies have found evidence relating tobacco consumption to the development of nephropathies in type 1 and 2 diabetes mellitus [4]. It has also been shown that the tobacco consumption is a risk factor for the development and progression of diabetic neuropathies [5].

According to recommendations in national and international guidelines, achieving smoking cessation is a priority in diabetic smokers, given that this group has two to four times more cardiovascular risk when compared to the general population [6-8].

In the present study, Prochaska and DiClemente's Transtheoretical Model of Change will be used. This model describes a series of successive stages (precontemplation, contemplation, preparation, action and maintenance) that signal a different type of intervention depending on the person's stage. Despite the discrepency regarding the utility of this model in smoking cessation [9,10], the majority of clinical guidelines in primary care (PC) consider useful because it helps to make decisions regarding treatment. Furthermore, it allows the professional to gauge the evolution of the patient's attitude towards smoking cessation.

Although there are differences between type 1 and type 2 diabetes, previous studies show that the majority of diabetic smokers are in the pre-contempative stage of Prochaska and DiClemente's model. In a study by Ruggiero et al, people that received some type of smoking cessation advice made more progress in the process of smoking cessation [11].

Cooximeters have been used to demonstrate continued smoking abstinence. Consensus documents have recommended this technique, concluding that it is useful in diagnosing and in aiding with the process of smoking cessation [12].

It its known that minimum advice for smoking cessation in the general population achieves a mean 5% smoking cessation in one year [13] while interventions with intensive follow-ups can achieve a mean of 20% smoking cessation [14]. In general, abstinence rates increase with the intensity of the intervention, the time used in the intervention and the number and diversity of contacts with the healthcare professional, including follow-up visits [4,15]. Health interventions in tobacco consumption can be considered to have a low cost in relation to the quality and years of life gained. From a public health point of view, the obtained results possibly constitute the most cost efficient process to improve the health of the population [14,16].

Integrating pharmocological treatments that have been shown to be efficient in meta analyses and systematic revisions (nictone substitutes, Bupropion and Varenicline) has also been shown to be useful [17-19].The efficacy and effectiveness of interventions using recommendations similar to the present study have also been studied in PC, such as the ISTAPS study [20].

Due to the difficulty of an interventon in a population as advised and resistant to change as diabetic smokers [21,12], advancing in the stages of change of Prochaska and DiClemente's model, as well as decreasing the number of cigarettes, are also seen as important in addition to smoking abstinence [21,12].Therefore, they will also be objectives in the current study.

Various studies have shown that the more intense an intervention, the better the results obtained. In Persson et al [22], a Swedish study carried out in 412 diabetic smokers in PC, subjects in the intervention group received smoking cessation advice and were followed by telephone or in group sessions, while subjects in the control group were mailed smoking cessation advice. The one-year abstinence rate was 20% in the intervention group and 7% in the control group. Furthermore, within the intervention group, 40% of those who participated in the group therapy quit smoking, compared to 14% in of those who were followed by telephone. In Canga et al [23], a randomized clinical trial that evaluated the effectiveness of an smoking cessation intervention using intensive follow-up in diabetic smokers, the six-month continued abstience rate was 17% in the intevention group and 2.3% in the control group. In intervention group subjects that did not quit smoking, the number of cigarettes smoked decreased. It was concluded that a structured intervention increases smoking cessation in diabetic smokers.

Another study in diabetic smokers carried out in Minneapolis, Minnesota [24] concluded that programs for diabetes education should include intensive interventions for smoking cessation because they increase the efficacy of advice and do not have a negative effect on the metabolic control of diabetes.

There are only a few studies published about smoking cessation in diabetic smokers in PC, despite the fact that most diabetic patients visit their PC physician various times per year for routine check ups. According to the World Health Organization [25], diabetes control and prevention activities should be based in PC. This suggests that PC is the ideal environment for intensive interventions in smoking cessation in diabetic smokers.

For all of the abovementioned reasons, the effectiveness of an intensive smoking cessation intervention in diabetic smokers in PC, based on the Prochaska and DiClemente Transtheoretical Model of Change and motivational interview techniques, will be evaluated in the present study.

## Hypothesis

The present smoking cessation intervention in diabetic patients in PC, which uses an intensive strategy that is motivational, individualized, structured and adapted accoding to the Prochaska and DiClemente's Transtheoretical Model of Change, will cause a difference in continued abstinence of 12% between patients that receive the intervention versus those who do not. This takes into consideration the fact that smoking cessation in the general population is 5% with minimum advice [13] and 20% with intensive advice [14].

## Objectives

### Principal objective

To evaluate the effectiveness of an intensive intervention to obtain continued smoking abstinence in diabetic patients in PC.

### Secondary objectives

1. To study the effectiveness of an intensive smoking cessation intervention in the evolution of Prochaska and DiClemente's Transtheoretical Model of Change in diabetic patients in PC.

2. To study the effectiveness of an intensive smoking cessation intervention in the evolution of tobacco consumption in diabetic patients in PC.

3. To quantify the time spent on the intensive smoking cessation intervention versus usual clincial practice in PC.

## Methods/Design

### Design

Cluster randomized trial, controlled and multicentric. Randomization unit: Primary Care Team (PCT).

### Setting

43 PCTs from the province of Barcelona that provide health coverage to urban, semirural and rural populations.

### Study population

Type 1 and 2 diabetic smokers older than 14 years of age that receive routine diabetes care in the participating PCT. An active smoker is defined as a patient that responds affirmatively to the following questions: 1. Do you smoke currently? 2. Have you smoked more than 100 cigarettes in your lifetime? 3. Have you smoked any tobacco product in the last seven days? [26,27].

### Exclusion criteria

Patients with communication difficulties (cognitive or sensory deterioration, language barrier); patients that have not had check ups for their diabetes in the PCT for more than one year; patients with terminal illnesses, serious psychologial illnesses, active addictions to other psychoactive substances; patients that are already in the process of quitting smoking; patients that live more than six months outside of the territory assigned to the PCT; and patients who do not consent to participate in the study.

### Withdrawl criteria

Any event that may lead to a situation that discourages the intervention or that may prevent communication with the healthcare professional.

### Sample size calculation

The sample size has been calcuated by multipying the size of a randomized simple design by the design effect. In the simple randomized design, considering an alpha error of 0.05 a beta error of 0.20 in a bilateral contrast, and given that 15% of diabetics are smokers, 124 subjects were needed in each study group in order to determine a difference in continued abstinence of greater than or equal to 12% between study groups. A continued abstinence of 5% was assumed for the non intervention group, and a potential loss of follow-up of 20% was estimated.

Using an intracluster correlation coefficient of 0.05 [28-30], and based on an average of 25 diabetic smokers per PCT, the design effect is 2.2. Therefore, 546 diabetic smokers and 22 PCT were needed. The sample size was calculated using the Granmo 5.2 program for Windows.

### Data Collection

#### Dependent Variables

• Continued abstinence (yes, no) is defined as at least six months without smoking and a CO breath level of <6 ppm, measured by a cooximeter in standard conditions.

• Stage change in Prochaska and DiClemente's Transtheoretical Model of Change (advance, maintained, backwards, relapse).

• Number of cigarettes per day, reported by the patient.

• Total time (in minutes) spent on the intervention.

#### Independent Variables

• Variables related to the patient (information obtained through an individualized interview):

- Administrative: PCT, PC professional, data collection date, patient code.

- Sociodemographics: date of birth, sex, level of education.

- Anthropometric: weight, height.

• Related to diabetes: type, year of diagnosis, type of treatment, presence of complications related to diabetes (peripheral arterial disease, ischemic heart disease, retinopathy, neuropathy, nephopathy).

• Related to associated pathologies: COPD, cerebrovascular accident, isquemic heart disease, hypertension, hypercholesterolemia.

• Variables related to tobacco consumption: age at initiation of habit, number of cigarettes per day, nicotine dependence (Fagerström test), motivation to quit (Richmond test), number of previous attempts to quit for at least 24 hours, smoking cessation treatment with mediciations, maximum time of abstinence, phase of the patient according to Prochaska and DiClemente's model (pre-contemplation, contemplation, preparation-action), tobacco in usual environment (family, partner, friends, coworkers, classmates), perceived grade of harmfulness of tobacco on diabetes, point prevalence abstinence (smoking abstinence at the time of the visit and with a CO level of less than 6 ppm), length of abstinence.

• Variables related to the intervention: attendance of intervention visit, date of intervention visit, date of D day, time spent on intervention visit, medication used for smoking cessation, relapse, reason for relapse, CO level in breath, observations.

• Variables related to the one-year follow-up visit: attendance to follow-up visit, date of follow-up visit, smoking status, number of cigarettes per day, length of abstinence, phase of the patient according to Prochaska and DiClemente's model (pre-contemplation, contemplation, preparation-action), CO level in breath, observations.

• Variables related to the PCT: years the PCT has been operating, assigned population, mean age of assigned population, whether or not it is a teaching center for residents, number of doctors, number of nurses, number of social workers, other specializations, number of centers.

### Description of the Study

The phases of the study are:

#### Phase 1

Recruitment of the PCT: The project was presented to all of the potential PCTs in order to recruit health professional that were interested in participating. Both doctors and nurses were allowed participate. Those who wanted to collaborate signed a comittment form.

#### Phase 2

Forming the study groups (Intervention group (IG) and non intervention group (NIG)) and training the health professionals.

- The unit of randomization was the center. The centers were assigned to either the IG o the NIG by simple randomization, with a ratio of 1 to 1. The system of randomization was centralized and computerized.

- The professionals in the IG received a specific training program that consists of a motivational interview workshop and a pharmocological treatment workshop specialized for smoking cessation. Both workshops were geared towards diabetic smokers and were taught by experts. The professionals also received training in how to use the electronic data collection system and in the dynamics of the follow-up visits according the Prochaska and DiClemente's Transtheoretical Model of Change. The professionals in the NIG attened practical training session that covered the methodology of the study and how to use the electronic data collection system.

#### Phase 3

Recruitment of the patients and initial categorization in IG and NIG (currently the study is in this phase):

- Selection of patients: Patients were recruited as they visited the PCT. If in the month prior to closing the patient recruitment phase a professional had not recruited the number of patients required, a list of diabetic smokers could be obtained and the necessary number was selected by simple randomization. The professional called the selected patients to make an appointment. According to a previous estimation and taking into consideration the sample size, each professional had to recruit five patients. The form of recruitment, as well as the number of patients to be recruited by each professional, was considered feasible for professionals in routine clinical practice.

- Inclusion of patients: If a patient met the inclusion criteria, the doctor or nurse explained the study to the patient and solicited the patient's participation. If the subject agreed, he or she received information about the study and signed a consent form. If the subject did not wish to participate, the motives were documented.

- Initial categorization of patients: In the first contact with the patient, the following variables were collected using an individualized interview.

- Independent: administrative, sociodemographic, anthropometric, those related with diabetes, associated pathologies and those related to the smoking habit

- Dependent: length of visit

- All of the data is being registered in the electronic data collection system which includes measures to assure consistency and quality control.

- Pilot program: A pilot program will be carried out in subjects that will be excluded from the study so as not to affect the final results. The pilot program aims to evaluate: the process of inclusion of subjects, data collection using the electronic system and the difficulties encountered in the intervention and in the established circuits.

#### Phase 4

**Figure 1:**
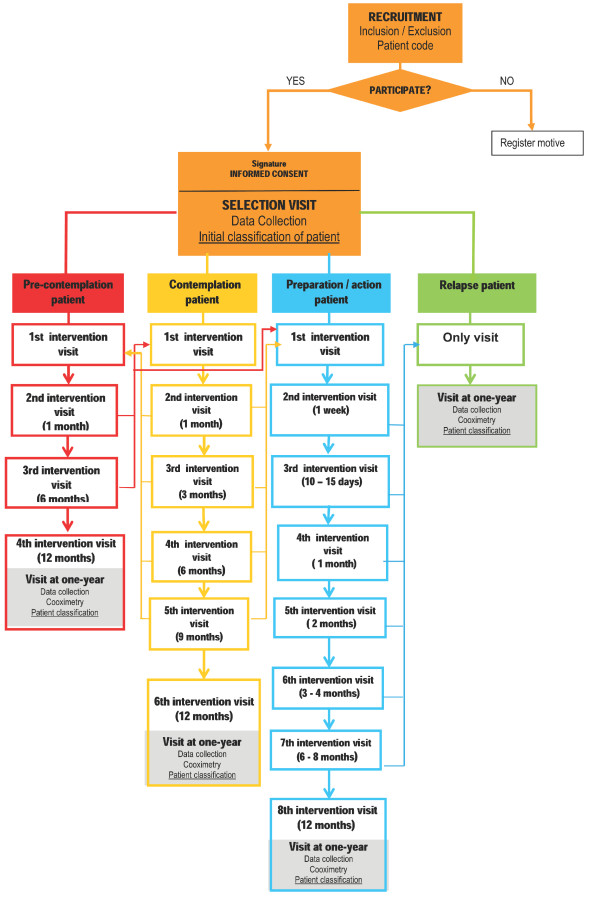
**ITADI Intervention group study algorithm**.

Intervention (only in IG): An intensive, individualized intervention using the motivational interview, conduct therapies and medications, adapted according to the stage of change of the patient, will be used. The number of visits will depend on the stage that the patient is in (five for pre-contemplation, seven for contemplation and eight for preparation-action). Patients can move forward and backward in their stage over the course of the study, and the study will adapt to these changes. The phases of the intervention will be detailed in the IG algorithm (see Figure 1).The variables to be collected are specified in the electronic data collection system.

The patients of the NIG group will receive the usual care in PC, and at the end of the study, the NIG professionals will also be trained in the motivational interview and in pharmocological treatments.

#### Phase 5

Final categorization (in IG and NIG): The dependent variables will be collected in all subjects at one-year post- inclusion. The motives for withdrawl and loss of follow-up from the study will be recorded and a flow chart of participants will be created over the course of the study.

#### Phase 6

End of data collection: Data collection will be concluded after carrying out the final patient categorization. Because of the periodic monitoring of the data in the electonic data collection system, incoherencies will have been located and corrected.

#### Phase 7

Data analysis and diffusion of results: The collected data will be analyzed, the final report will be elaborated and the results will be diffused in various scientific outlets.

### Statistical analysis

Data will be analyzed in concordance with the Consort Cluster guide [31], and all analyses will be done on an intention to treat basis. A descriptive analysis and an analysis of baseline comparability between the study groups will be performed for the variables studied. A T-student test or a U Mann Whitey will be used to compare means with two categories, an ANOVA test will be done to compare means with two or more categories, and a Chi- squared test or a Chi -squared tendency test will be done to compare categorical variables. A multilevel logistic regression and a Poisson regression will be done to evaluate the association between each of the dependent variables and the independent variables that have resulted statistically significant in the bivariate analisis. All analisis will be adjusted for potential confounding factors and for variables with clinical relevance. All tests will be done with a 95% bilateral confidence interval. SPSS 15, STATA 10 and HLM6 will be used.

### Quality Control

Several procedures were employed to ensure the quality of the study data, thus maximizing the validity and reliability of the program delivery and outcome assessments. They were:

• An eight -hour training the professionals who participated in the study in motivational interviewing, pharmocological treatment of smoking, and use of the electronic data collection system.

• Use of an electronic data collection system with a system of warnings and intenal coherency noms.

• A full time person contracted to provide technical and methodological support to the study participants.

• Regular meetings and mailings between members of the study group and all participating centers.

### Ethical Aspects

In the first contact with the patient, he or she will be given information about the study and will sign an informed consent form if he or she wishes to participate. The informed consent will detail the ethical conditions and the paricipant's right to intimacy, anonymity, confidentiality, withdrawl and information. The researchers also commit to respect the norms of good clinical practice, as well as the requirements of the Helsinki Declaration. The protocol has been evaluated and approved by the Ethical Committee of Scientific Reseach and the Jordi Gol Center for Investigation in Primary Care. Confidentiality of data: Only the investigators and monitors/auditors of the study will have access to the data of the subjects who agreed to participate.

## Discussion

Although there are studies that use intensive interventions in smoking cessation such as the ISTAPS study [20], the majority are directed towards the general population. The challenge of the present study is to exclusively direct the intervention towards diabetic smokers in PC. The fact that these type of patients are resistant to change should not be an obstacle, as it is a priority to address the topic of smoking when dealing with patients with an elevated cardiovascular risk. Furthermore, it should be kept in mind that the intention of this intervention is not only to achieve continued smoking abstinence, but also to achieve partial goals, such as advancing in the stages of change of Prochaska and DiClemente and the reduction of the number of cigarettes smoked per day. These partial goals can motivate a healthcare professional who has tried unsuccessfully on several occasions to get his or her patient to quit smoking, to continue working towards progress.

One of the stengths of the ITADI study is the population of the study, diabetic patients that attend PC. Another strength is the design of the study, which is pragmatic in terms of time and material resources needed. If the hypothesis of the present study is confimed, it could be incorporated into current PC clinical practice guidelines. The large number of healthcare professionals and patients involved in the present study is another strength, as it reinforces the external validity of the data.

The randomization has been done by PCT and not by patient, so as to avoid the unethical situation in which a healthcare professional would feel obligated to use the motivational interview and pharmocotherapy in only some of his or her patients. This will also prevent contamination between healthcare professionals of the same center. Although the unit of randomization is the center, the intervention is given at the individual level and the outcomes are also measured at this level. The estimation of the design effect will be taken into account in all of the main analyses.

The motivational interview techiques require personal communication and empathy from the part of the healthcare professional towards his or her patient. The training sessions are one full day, although it is known that the more training the professional has in motivational interviewing, the more he or she is able to apply the learned skills [32].

Cooximetry is a technically simple and affordable method to apply in PC, as well as valid and useful [12]; however, it has some management limitations. In order to overcome these limitations, the healthcare professionals of both study groups will be trained in how to use cooximeters. Furthermore, an instruction manual and audiovisual guide will be designed on how to use the cooximeters. The cooximetry measures will be taken in the afternoon in homogenous and standarized conditions.

Finally, the current study will try to describe the profile of a diabetic smoker who receives the most benefit from an intensive smoking cessation intervention in PC. The goal is to use the results of the study as a foundation for a protocolized intervention in PC guidelines for diabetes

## Competing interests

The authors declare that they have no competing interests.

## Authors' contributions

LR, SP, CM, GP participated in the revision of the bibliography and design of the study methodology. SP formulated the study question. LR and MA contributed in the writing and translation of the present manuscript. The members of the ITADI Group are participating in recruitment and follow-up of the patients. All of the authors have read and approved of the present manuscript.

## Pre-publication history

The pre-publication history for this paper can be accessed here:

http://www.biomedcentral.com/1471-2458/10/58/prepub

## Aknowledgements

This study was carried out with financial help from an Evaluation of Sanitary Technologies and Health Services grant (Evaluación de Tecnologías Sanitarias y Servicios de Salud), given by the Carlos III Health Institute (PI08/90345) in 2008.

The authors appreciate the support from the Unit of Research Support of Metropolitan North and the Unit of Research Support of Barcelona and. Nodo Cardiocat (Research Network on Preventive Activities and Health Promotion -G06-128). Furthermore, they thank IDIAP Jordi Gol, in particular Teresa Rodriguez and Cecilia Borau.

The authors would also like to thank the Catalan Institute of Health and Health Consortium of Terrassa, PANEM (Prestaciones de Asistencia Médica al Personal Municipal), as well as their respective centers of employment (ABS La Llagosta. ABS La Garriga, ABS Passeig Sant Joan, ABS Badia, ABS Terrassa Nord), for allowing them to attend numerous design and coordation meetings for the study.

The authors also thank all of the associated researchers of the ITADI Group that have made the development of the field work possible.

Associated researchers:

Ma Eugèni Adzet, Jose Javier Alba, Enriqueta Alcaraz, Immaculada Alegre, Montserrat Alegret, Carolina Alejo, Jordi Alfonso, Carles Alonso, Carmen Alonso, Cristina Alós, Judit Alsina, Ma Montserrat Alsina, Albert Alum, Paula Álvarez, Marta Amatller, Paloma Amil, Eulàlia Andreu, Rosa Ma Andreu, Antonio Aranzana, Melanie Ardite, Eva Ares, Montserrat Argila, Maria Jesús Arroyo, Joan Assens, MariaAtero. Rita Ayala, Gema Badell, Alicia Balsells, Esther Barberan, Anna Barceló, Montserrat Baré, Alex Bassa, Carme Batalla, Noemi Beatriz, Joan Begué, Esperanza Bello, Ma Antònia Benedi ,Ma Rosa Benedicto, Yolanda Bermejo, Jesus Bernad, Nuria Bertolin, Maite Bertran ,Begoña Bessa , Alesandro Bilardi, Paola Blanco, Lidia Bombo, Elena Bonilla ,Sandra Bosch, Ana Ma Bravo, Maria Luisa Bravo, Montse Brugada, Montserrat Bulnes, Alicia Burgos, Inmaculada Burgos, Raquel Buron, Teresa Cabello, Rosa Cabello, Ana Calderón, Ana Calonge,Francisco Javier Calpe, Ana Callejero, Xavier Camarena ,Josepa Canadell, Gemma Canals, Ricard Canals, Raquel Cano, Erika Cano, Silvia Cárceles , Claudia Cardoner, Jose Manuel Carmona, Montserrat Carol, Montse Carrasquer, Remedios Carreño,Dolores Carrillo, Miquel Casadevall,Ramon Casals,Gemma Casanovas, Isabel Casas, Ana Cascos, Miriam Ceña, Ana Clara Cereijo, Rosario Ciganda, Miquel Cirera, Magi Claveria, Carme Clopés, Anna Clot, Sònia Codina, Glòria Colomé, Araceli Comas, Mo Carmen Conde, Toni Contreras, Antonia Correas, Imma Corretjé, Gemma Cortés, Dolors Costa, Laura Cristel, Silvia Crivillé, Jose Manuel Cruz, Maria Isabel Cuenda, Joaquina Cuevas, Judith Cuspinera , Marta Chaves, CristinaDapena, Ma Dolors Daura, Rosa De la Iglesia, Ma Amparo De la Poza, Cristina Delso, Trinidad Diaz, Fernando Diaz , Alicia Díaz, Ma Amparo Diez, Joan Dordas, Joan Elias, Gerard Escriche, Ma Teresa Esteban, Jose Luis Estebanez, Rosa Ma Esteve, Gema Estrada, Marc Ezcurra, Anna Fabra, Eva Fábrega, Gemma Fanlo, Josep Feliu, Juan Fdo Fernandez, Teresa Fernandez, Esther Fernandez, Jorge Fernández, Belén Fernández, Josefina Fernández,Antonia Ferre, Montserrat Ferrer, Mo del Mar Ferrero, Inmaculada Figueras, Souhel, Flayeh, Susana Florentí, Teresa Font, Ma José Font, Didac Fores, Martí Fradera, Jordi Gago, Nuria Garcia, Manel Garcia, Elisenda Garcia, Carlos Garcia, Concepcion Garcia, Carme García, Marta García, Isabel García, Eva García, Marina Garrel, Patricio Garrido, Isabel Garro, Veronica Gasque, Griselda Gasulla, Isabel Gavin, Marta Gelpí, Jana Gerhard, M. Àngels Gibert, Silvia Giménez ,M. Eugenia Giménez, Montserrat Giné, Anna Giné, Alfonso Girona, Carmen Gómez, Ana Gómez-Quintero, Esther Gonzalez, Almudena González, Begoña González, Cristina González, Concepción Gonzàlez, Maria Gordillo, Sergi Granell, Francisco José Granero, Silvia Granollers, Elisenda Guarné, Isabel Guerrero, Carolina Guiriguet, Mercè Guiu, Agustí Guiu, Juana Gutierrez, Felipe Maria Hernandez, Eustaquio Hernandez, Enric Hernandez, Rosa Ma Hernandez, Eva Hernández., Maria del Mar Herranz, Marta Herranz, Juan Herreros, Ma Gloria Huerta, Carme Iglesias, Carmen Ioana Jarca, Núria Jarque, Conxita Jordán, Purificación Jordana, Teresa Jose Maria, Azucena Lara, Miriam Lara,Mónica León, Manuela Licerán, Mercedes Liroz ,Isabel López, Victor Miguel López, Rosa Maria López, Aurora López, Cristina López, Mo Rosa López, Cristina López, Virtudes López, Maite López. Ma Begoña López. Maite Lorén. Ma Carmen Losada, MaribelLozano, Roque Lucas, Lorena Luengo, Carmen Llamas, Mercedes Llorente, Immaculada Malé, Regina Malfeito, Susana Manero, Ma José Manzano, Blanca Mañé, Maria José Marco, Montse Marles, Jordi Martí, Nuria Martí, Mo Teresa Martí, Maria Benita Martín, Juan José Martín, Ma Gloria Martinez, Esther Martínez, Ana Martínez, Fèlix-Salvador Martínez, Rafael Martínez, Ma Sierra Martínez, Xavier Martínez, Miriam Martínez, Milagros Martínez, Javier Martos, Mercè Mas, Imma Mas, Meritxell Masip, Carme Mata, Atanasio Mata, Francesca Mata, Marisol Mayorga, Rafael Medrano, Rosa Melero, Roser Melgar, Estebana Mendoza, Jordi Mestre, Ma Teresa Millán, Joan Mogas, Ma Lluïsa Molina, Gemma Molina, Marta Molina, Ma Jesus Molina, Eva Mon, Ericka Montalvo, Isabel Montaner, Meritxell Montasell, Montserrat Montasell, Ma Remedios Montes, Laura Montes, Susanna Montesinos, Ana Montoliu, Susana Mora, Amparo Moraga, Sandra Moraleda, Beatriu Moreno, Juan Andrés Moreno, Gema Mota, Carlos Luis Mota, Mari Fe Muñoz, Mo José Narvaez, Montserrat Navarro, Carmen Navarro, M. Paz Nieto, Laura Nieto, Montserrat Ochando, Jose Ignacio Olivares, Josep Oliveras, Sonia Omella, Pilar Orellana, Julià Ortega, Raquel Ortega, Sergi Ortiz, Eva Pablos, Encarna Pachon, Jesús Pagés, Lidia Palau, Carme Pallarés, Carme Pallarés, Daniel Pañart, Carme Pascua, M. Encarnación Pastor, Marta Paytubi, Marta Pedrol, Ma Dolores Peleato, Xavier Peral. Yolanda Perez. Edurne Pérez, Montserrat Pérez, Juan Antonio Pérez, Ana Maria Pérez, David Pérez, M. Teresa Pérez, Ma José Pérez, Ana Maria Pérez del Molino, Maria P-Hervada, Mar Pinos, Lucio Pinto, Núria Piquer, Ma Antònia Pons, Alex Prats, Josep Puig, Ma Luisa Puigcerver, Francesc Pujol, Jordi Pujol, Cecilia Quer, Amelia Quílez, Núria Rafols, Concepcion Ramírez, Elisenda Realp, Estibaliz Redondo, Anna Reixach, Montserrat Reverté, Isabel Rey, Ma Begoña Ribas, Ana Maria Ribatallada,Gema Rico, Maria Riera, Lydia Riera, Sofia Riu, Carles Rius, Nuria Rivero, Agueda Robles, Ma Teresa Rodellar, Jose Luis Rodenas, Inés Rodríguez, Vanesa Rodríguez, Isabel Rodríguez, Mònica Rodríguez, Silvia Roig, Ma Isabel Román, Fina Romanyà, Mireia Rosàs, Montserrat Roviralta, Mariano Rozas, Marta Ruberte, Eva Rubio, Felisa Rubio, Ma Teresa Ruiz, Mercè Ruiz, Sonia Ruiz, Olga Ruiz, Josep Maria Ruiz, Fernando Rupérez, Mo Mar Rus, Victòria Sabaté, Juan Antonio Sabio, Maria Assumpció Sagarra, Fina Sala, Fara Sanchez, Mo Belen Sanchez, Marta Sanchez, Sònia Sánchez, Carolina Sánchez, Roger Antonio Sánchez. Sara Sancho, Anselma Sandin, Ana Maria Santín, Gisela Sanz, Nieves Sanz, Alejandro Serra, Josefina Servent, Susana Sevillano, Gemma Simon, José Francisco Sobrino, Lourdes Sodric, Ma Pilar Solé, Merce Soler, Ma Luisa Sole, Esther Soria, Yolanda Suárez, Ana Maria Subias, Francesc Tarrés, Maria Pilar Tejero, Encarnació Tor, Olga Torner, Montse Torra, Mo Lourdes Torrabadella, Nativitat Torres, Araceli Torrubia, Jordi Tost, Ana Turnés, Esther VacasMa Carme Vallduriola, Antonio Vallejo, Pilar Vazquez, Inmaculada Vázquez, Assumpció Vàzquez, Ramon Vecayos, Rosa Ma Verdú, Concepció Vergara, Montserrat Vidal, Angels Vidaña, Ma Angela Vila, Rosa Vila, Carme Vila, Ramon Vilà, Imma Vilafranca, Maria Pilar Vilagrasa, Anna Vilaseca, Florencia Villanova, Dosinda Villanueva, Ma Carmen Viñes, Asunción Wilke, Carolina Yeste, Maria Dolors Ylla, Maria Vega Zafra, Monica Zambrano
